# Monitoring and surveillance for multiple micronutrient supplements in pregnancy

**DOI:** 10.1111/mcn.12501

**Published:** 2017-12-22

**Authors:** Zuguo Mei, Maria Elena Jefferds, Sorrel Namaste, Parminder S. Suchdev, Rafael C. Flores‐Ayala

**Affiliations:** ^1^ Division of Nutrition, Physical Activity and Obesity, National Center for Chronic Disease Prevention and Health Promotion Centers for Disease Control and Prevention (CDC) Atlanta GA USA; ^2^ Strengthening Partnerships, Results, and Innovations in Nutrition Globally (SPRING) Arlington VA USA; ^3^ Helen Keller International New York City NY USA

**Keywords:** monitoring and surveillance, multiple micronutrient, pregnancy, supplementation

## Abstract

The World Health Organization (WHO) recommends iron–folic acid (IFA) supplementation during pregnancy to improve maternal and infant health outcomes. Multiple micronutrient (MMN) supplementation in pregnancy has been implemented in select countries and emerging evidence suggests that MMN supplementation in pregnancy may provide additional benefits compared to IFA alone. In 2015, WHO, the United Nations Children's Fund (UNICEF), and the Micronutrient Initiative held a “Technical Consultation on MMN supplements in pregnancy: implementation considerations for successful incorporation into existing programmemes,” which included a call for indicators needed for monitoring, evaluation, and surveillance of MMN supplementation programmes. Currently, global surveillance and monitoring data show that overall IFA supplementation programmes suffer from low coverage and intake adherence, despite inclusion in national policies. Common barriers that limit the effectiveness of IFA—which also apply to MMN programmes—include weak supply chains, low access to antenatal care services, low‐quality behaviour change interventions to support and motivate women, and weak or non‐existent monitoring systems used for programme improvement. The causes of these barriers in a given country need careful review to resolve them. As countries heighten their focus on supplementation during pregnancy, or if they decide to initiate or transition into MMN supplementation, a priority is to identify key monitoring indicators to address these issues and support effective programmes. National and global monitoring and surveillance data on IFA supplementation during pregnancy are primarily derived from cross‐sectional surveys and, on a more routine basis, through health and logistics management information systems. Indicators for IFA supplementation exist; however, the new indicators for MMN supplementation need to be incorporated. We reviewed practice‐based evidence, guided by the WHO/Centers for Disease Control and Prevention logic model for vitamin and mineral interventions in public health programmes, and used existing manuals, published literature, country reports, and the opinion of experts, to identify monitoring, evaluation, and surveillance indicators for MMN supplementation programmes. We also considered cross‐cutting indicators that could be used across programme settings, as well as those specific to common delivery models, such as antenatal care services. We then described mechanisms for collecting these data, including integration within existing government monitoring systems, as well as other existing or proposed systems. Monitoring data needs at all stages of the programme lifecycle were considered, as well as the feasibility and cost of data collection. We also propose revisions to global‐, national‐, and subnational‐surveillance indicators based on these reviews.

**Key messages**

- Monitoring and surveillance is a key component of multiple micronutrient (MMN) supplementation in pregnancy.
- A set of indicators across the programme lifecycle are proposed for consideration when designing an MMN supplementation monitoring and surveillance system. Many of the indicators are modifications of existing iron‐folic acid (IFA) supplementation indicators with the addition of outcome indicators afforded by MMN supplementation.
- At a minimum, most programmes should include indicators related to the monitoring of supply, coverage, and intake adherence. Policy indicators are also critical when shifting from an IFA to MMN supplementation programme.
- Development of a feasible and cost effective quality of services indicator is needed.

## 1 INTRODUCTION

Iron deficiency remains the most common micronutrient deficiency worldwide, particularly affecting children and women of reproductive age, including pregnant women (http://www.who.int/nutrition/topics/ida/en; Kassebaum et al., 2014; Stevens et al., 2013). The World Health Organization (WHO) currently recommends a daily dose of 30–60 mg elemental iron (60 mg preferred in setting where anaemia prevalence >40%) and 0.4 mg folic acid taken as early as possible in pregnancy to improve maternal and infant health outcomes (World Health Organization, 2012; World Health Organization, 2016). Continued supplementation beyond pregnancy is also recommended if iron deficiency prevalence in the country is high, or if the pregnant woman is anaemic (Stoltzfus & Dreyfuss, 1989). Iron and folic acid (IFA) supplementation during pregnancy reduces anaemia and iron deficiency at term, as well as the risk of low birth weight (Peña‐Rosas, De‐Regil, Dowswell, & Viteri, 2012). Other micronutrient deficiencies, beyond iron and folate deficiency—such as vitamin A, vitamin D, vitamin B_12_ and zinc—are also widely prevalent among women of reproductive age, particularly pregnant and lactating women in developing countries (Bhutta et al., 2013; Dalmiya, Darnton‐Hill, Schultink, & Shrimpton, 2009; Jiang, Christian, Khatry, Wu, & West, 2005; World Health Organization, 1992). Poor maternal nutrition is frequently caused by poor access to nutrient‐adequate foods, cultural practices that discourage women from gaining weight, long hours of physical labour, and recurrent infections (Christian, 2010). In 1999, the United Nations Children's Fund (UNICEF), United Nations University (UNU), and WHO agreed on the composition of a proposed multiple micronutrient (MMN) tablet called United Nations International Multiple Micronutrient Preparation, which provides the recommended daily allowance of vitamin A, vitamin B1, vitamin B2, niacin, vitamin B6, vitamin B12, folic acid, vitamin C, vitamin D, vitamin E, copper, selenium, and iodine with 30 mg of iron and 15 mg of zinc for pregnant women (UNICEF/UNU/WHO, 1999). Efficacy and effectiveness trials have confirmed that MMN supplementation during pregnancy can improve outcomes beyond anaemia, including deficiencies of other vitamins and minerals, low birth weight, small for gestational age (Dalmiya et al., 2009; Fall, Fisher, Osmond, & Margetts, 2009), and preterm birth (West et al., 2014). A recent Cochrane review of MMN supplementation in pregnant women assessed 17 trials and reported that MMN resulted in a significant decrease in the number of newborn infants identified as low birthweight (risk ratio [RR] 0.88, 95% confidence interval [CI; 0.85, to 0.91]) or gestational age (average RR 0.90, 95% CI [0.83, 0.97]), and a reduced rate of stillbirth (RR 0.91, 95% CI [0.85, 0.98]; Haider & Bhutta, 2015). There were no significant differences for other maternal and pregnancy outcomes such as preterm births, maternal anaemia in the third trimester, miscarriage, maternal mortality, perinatal mortality, neonatal mortality, or risk of delivery via a caesarean section (Haider & Bhutta, 2015). Though the evidences were with insufficient data on neurodevelopmental and long‐term follow‐up of maternal and offspring outcomes, these findings still support the potential replacement of IFA supplements in pregnancy with MMN supplements in populations at risk (Bhutta et al., 2013).

**Figure 1:**
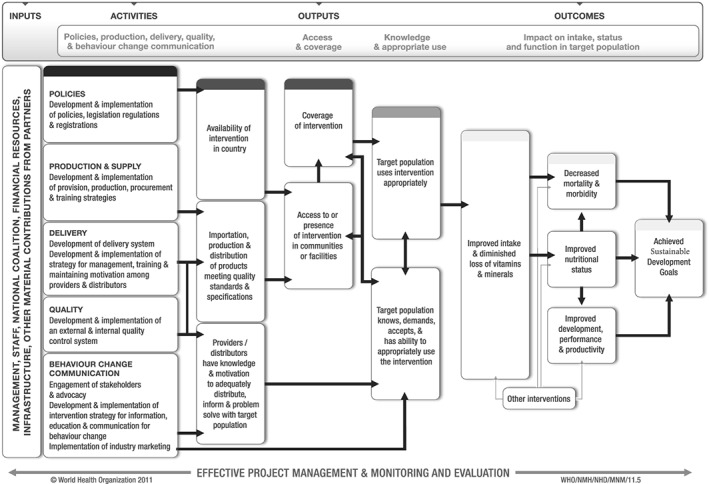
WHO/CDC logic model for vitamin and mineral interventions in public health programmes (De‐Regil et al., 2014)

According to the WHO/Centers for Disease Control and Prevention (CDC) logic model for vitamin and mineral intervention in public health programmes (De‐Regil, Peña‐Rosas, Flores‐Ayala, & Jefferds, 2014), most micronutrient programmes are expected to follow particular intervention processes. These processes require inputs to support relevant policies, adequate production and supply, delivery of the programme, quality control, and behaviour change communication and intervention strategies in order to achieve expected outputs and outcomes (Figure 1). All programmes also require effective programme management and monitoring. However, global surveillance data show that IFA supplementation programmes often suffer from low coverage and intake adherence of supplements, despite many countries include supplementation during pregnancy in national policies. Common barriers limiting the effectiveness of these programmes, which would also apply to MNP supplementation programmes, include weak supply chains, low access to antenatal care (ANC) services, gastrointestinal side effects, and poor quality interactions between healthcare providers and women, low‐quality behaviour change interventions to support and motivate women, and weak or non‐existent monitoring systems used for programme improvement (Bhutta et al., 2009; Deitchler, Mathys, Mason, Winichagoon, & Tuazon, 2004; Galloway & Mcguire, 1994; Nguyen et al., 2008; Sununtnasuk, D'Agostino, & Fiedler, 2015; Tessama, Jefferds, Cogswell, & Carlton, 2009; WHO, 2012). The causes of these barriers in a given country require careful review and appropriate resource allocation to be resolved.

National and global monitoring and surveillance data on supplementation during pregnancy are primarily collected from cross‐sectional surveys and on a more routine basis through health and logistics management information systems. Some indicators for IFA supplementation exist (Bhutta et al., 2009; Tessama et al., 2009); however, based on the WHO/CDC logic model (De‐Regil et al., 2014), new indicators for MMN supplementation need to be incorporated, especially around policy development and inclusion of additional outcomes afforded by MMN compared to IFA supplements. Identifying key monitoring indicators that address barriers and support effective programmes is a priority as countries heighten their focus on supplementation during pregnancy and may potentially initiate or transition to MMN supplementation.

In 2015, WHO, UNICEF, and the Micronutrient Initiative (MI) held a “Technical Consultation on MMN supplements in pregnancy: implementation considerations for successful incorporation into existing programmemes,” which included a call for indicators needed for monitoring, evaluation, and surveillance of MMN supplementation programmes. The objective of this paper is to describe monitoring, evaluation, and surveillance indicators for MMN supplementation in pregnancy programmes.

## 2 METHODS

### 2.1 Principles used to guide the selection of indicators in monitoring and surveillance

Monitoring is the ongoing process of collecting, analysing, interpreting, and reporting indicators, to compare how well a programme is being executed against expected objectives (Home Fortification Technical Advisory Group, 2013). Nutrition programme monitoring is a continuous assessment and oversight of the nutrition programme's implementation stage. The purpose is to determine if the planned programme activities and expected outputs have been achieved; and, if not, what actions can be taken to quickly correct problems.

Public health surveillance is the ongoing, systematic collection, analysis, interpretation, and dissemination of data for a health‐related event that will be used in public health action to reduce morbidity and mortality and to improve health (CDC 2001; CDC 1999). Nutrition surveillance is the ongoing, systematic collection, analysis, interpretation, and dissemination of data regarding nutritional status and nutrition programmes to make policy and programmatic decisions that will lead to improvements in the nutrition situation of a population. While national nutrition surveillance focuses on programmes in an individual country, global surveillance examines the types and number of nutrition programmes and key indicators, such as coverage in countries around the world.

Logic models are useful tools to structure and organize information, and they can be used to convey information and expected processes in ways that are simple or complex. The WHO/CDC logic model for micronutrient interventions in public health (Figure 1) is a tool to assist countries in designing and implementing micronutrient programmes that are focused on achieving public health goals (De‐Regil et al., 2014). The logic model is organized according to four main hierarchical categories: inputs, activities, outputs, and outcomes (De‐Regil et al., 2014). We used the WHO/CDC logic model (Figure 1) as a template to visualize the expected intervention processes for the selection of indicators and to identify high‐priority indicators based on the review of practice‐based evidence and the sources mentioned above.

### 2.2 Process for selecting indicators

Guided by these definitions of monitoring and surveillance, we reviewed practice‐based evidence including monitoring manuals and guidelines, implementation manuals, and country reports from public health supplementation programmes; and expert opinion to identify existing and potential programme monitoring and evaluation indicators and potential data sources. In addition, we reviewed published literature, including literature related to micronutrient supplementation on monitoring and evaluation; efficacy trials and effective ANC and micronutrient supplementation programmes; cost; and systematic reviews on micronutrient supplementation. We also contacted experts in the areas of micronutrient supplementation implementation, ANC, and programme monitoring and evaluation. Programme input, activity, and output indicators were primarily found in guidance documents, whereas outcome indicators (e.g., related to status) were generally found in published literature. To identify global surveillance indicators and potential data sources, we examined questions included in the Demographic Health Surveys (DHS), Multiple Indicator Cluster Survey (MICS), and national nutrition surveys as these surveys generally collect the most prioritized indicators requested for global reporting and are used for cross‐country comparison. The selection of indicators was also guided by the WHO/CDC logic model and input, activity, output, and outcome categories.

Potential indicators were categorized by the boxes in Figure 1 for the monitoring and surveillance of MMN supplementation in pregnancy. First, we developed a list of indicator titles based on the WHO/CDC logic model that cover the areas of inputs, activities, outputs, and outcomes. This initial list was reduced to the final list by taking into consideration programme lifecycle, feasibility, and cost. For example, we do not list any indicators for the outcome box “Improved development, performance, and productivity” as they are complex and costly to collect and are not needed for most programme context. Second, we expanded the indicator titles into an indicator matrix so that each indicator title included a description of the type of indicator (e.g., input), calculation of the indicator or operational definitions, and potential data collection methods or data sources, suggested frequency and timing of data collection, and possible targets for some indicators. Finally, we identified a set of key indicators for different public healthcare settings.

## 3 RESULTS

### 3.1 Summary of proposed indicators for MMN supplementation programmes

Table 1 presents the proposed indicator titles for the MMN supplementation monitoring and surveillance systems, including inputs, activities, outputs, and outcomes. We proposed indicators for the corresponding WHO/CDC logic model (De‐Regil et al., 2014) box categories. Overall, Table 1 includes 30 potential input, activity, output, and outcome indicators that can be selected and adapted to any setting or specific programme depending on resources and the stakeholder and programme needs and priorities. Appendix A presents the indicator matrix for the 30 potential indicators identified from the literature and the expert panel, including descriptions of the type of indicator, calculation of the indicator or operational definition, potential data collection methods or data sources, suggested frequency and timing of data collection, and the potential expected target for each indicator. Some indicators are further divided into two or three subindicators to fully capture the programme's progress and impact.

**Table 1:** Indicator titles by relevant indicator types and logic model categories for consideration in the monitoring and surveillance of multiple micronutrient (MMN) supplementation in pregnancy programmes

Type of indicator	Corresponding WHO/CDC logic model (CDC 2001) box category	Indicator title
Inputs	Input: management, staff, national coalition, financial resources, infrastructure, other material contributions from partners	Key stakeholders support MMN supplements in pregnancy programme
		Work plan for forthcoming year confirms supplies, human resources, and budget
		MMN supplement‐related management and coordination group with defined responsibilities
Activities	Policies	National policy/guideline in place includes MMN supplements in pregnancy
	Production and supply	Work plan exists for timely procurement of supplements
		Training materials and job aids developed
	Delivery	MMN distribution system in place
		Supportive supervision system developed
	Quality	Quality assurance plan in place for MMN supplement programme
	Behaviour change communication	Behaviour change intervention strategy exists for MMN supplementation focused on supporting programme participants and programme advocacy
	Availability of intervention in country	MMN supplements available at the national or centralized‐level warehouse
	Importation, production, and distribution of products meets quality standards and specifications	Certificate of conformity for each shipment of imported MMN supplements
		Adequate storage of MMN supplements at warehouse and distribution sites
		All distribution sites submit timely reports per national guidelines
	Providers/distributors have knowledge and motivation to adequately distribute, inform, and problem‐solve with target population	Timely training (initial and refresher) activities conducted for providers/distributors per national guidelines
		MMN supplementation providers/distributors meet minimum knowledge criteria
Outputs	Coverage of intervention	Coverage of MMN supplements among pregnant women
		Coverage of counselling on MMN supplementation among pregnant women
	Access to or presence of intervention in communities or facilities	Sufficient MMN supply at distribution sites to cover the target population
	Target population knows, demands, accepts and has ability to appropriately use the intervention	Skills, knowledge, motivation, and attitudes about MMN supplementation among pregnant women, family decision makers, and community leaders
	Target population uses intervention appropriately	MMN supplement intake adherence among women during pregnancy
Outcomes	Improved nutritional status	Iron status, as measured by serum or plasma ferritin
		Folate status, as measured by red blood cell folate
		Anaemia status, as measured by haemoglobin
		Iodine status, as measured by urinary iodine concentrations
	Decreased morbidity and mortality	Low birth weight
		Small for gestational age
		Birth prevalence of neural tube defects
		Pregnancy‐induced hypertension
		Maternal mortality

### 3.2 Key indicators for MMN supplementation programme monitoring

Table 1 includes a comprehensive list of indicator titles for countries to consider when designing monitoring systems for their MMN supplementation programmes. Based on practice‐based evidence and expert opinion, there are three indicators that most countries should monitor at a minimum. An indicator on sufficient MMN supplement supply at distribution sites to cover the target population and avoid stock outs is critical because a well‐functioning supply chain is an essential component of any effective supplementation programme. Coverage of MMN supplements among pregnant women and intake adherence are the other two key indicators for assessing the success of MMN supplementation programmes. Coverage is defined as the percentage of pregnant women who receive the MMN supplements according to national policies or guidelines. Intake adherence is the percentage of pregnant women who consume the MMN supplements in the specified amounts according to national policies or guidelines. Adequate MMN coverage and intake adherence are the final results (outputs) of a well‐functioning MMN supplement supply and distribution system, adequate quality control, and robust behaviour change communication.

Routine data from Ministry of Health logistics management information systems (LMIS) and health management information systems (HMIS) are the most common sources of MMN supplement stock (commonly collected by LMIS) and distribution/coverage information (commonly collected by HMIS). These data systems provide information on a continuous basis (e.g., monthly) at lower administrative levels and are routinely forwarded to district/province and central levels. However, the current LMIS and HMIS systems in most developing countries are often weak and do not provide adequate quality data for assessing and guiding health programmes (Hancioglu & Arnold, 2013). Nationally representative, high‐quality household surveys, such as the DHS and MICS, play an important role in assessing coverage and intake adherence.

### 3.3 National and global surveillance of MMN supplementation programmes

The DHS, MICS, and national nutrition surveys are a common source of data for national and global surveillance. Currently, they typically include standard indicators related to supplementation programmes focused on recent coverage and intake adherence and often use similar wording whether asking about MMN or IFA supplementation. Two questions are asked in the DHS Phase 7 (2013–2018) women's questionnaire related to iron supplementation during a woman's last pregnancy in the previous 5 years: coverage (Q.420: During this pregnancy, were you given or did you buy any iron tablets or iron syrup?) and consumption/intake adherence (Q.421: During the whole pregnancy, for how many days did you take the tablets or syrup?).

In 2014, in response to the DHS call for suggestions to modify their core questionnaire, the CDC, Strengthening Partnerships, Results, and Innovations in Nutrition Globally (SPRING), and MI suggested the following revisions to replace these two questions with a set of six questions. The questions would improve our understanding of where women obtain supplements, the formulation they take (e.g., iron, IFA, or MMN), whether the supplements were obtained free of charge or were paid for, and how many tablets were received during the pregnancy. It was suggested that Q.420 be revised into five questions (Q.420a. During this pregnancy, were you given or did you buy iron or IFA tablets, iron syrup, or multiple micronutrients? Q.420b. If more than one form was received or purchased, in which form did you receive or purchase the most? Q.420c. Did you get iron tablets, iron syrup, or multiple micronutrients during an antenatal care visit, during another visit to a health facility, at a pharmacy, from a community worker/volunteer, or from another source? Q.420d. Did you purchase your iron or multiple micronutrients supplements or receive it free of charge? Q.420e. During the entire pregnancy, how many iron/IFA tablets, syrup, or multiple micronutrient supplements did you receive or purchase [all forms; iron/IFA tablets, syrup, and multiple micronutrients]?) It was recommended that Q.421 be revised, too (During the entire pregnancy, how many iron/IFA tablets/syrup or multiple micronutrient supplements did you take?). We plan to continue to work closely with DHS to consider these revisions in future iterations of the DHS survey.

SPRING has developed a simple method to use DHS data to make a rapid, initial assessment of the distribution and consumption of IFA supplements among pregnant women (Sununtnasuk et al., 2015). Briefly, this method assesses four sequential points at which an IFA supplementation programme commonly falters: ANC attendance/coverage, receipt of at least one IFA tablet, consumption of one or more IFA tablets, and the consumption of the ideal number of IFA tablets. This information can be used to identify strong and weak components of a countries supplementation programme and inform areas where formative research or context assessments should be conducted to determine the causes of faltering performance and identify solutions. The application of this tool in national planning has yet to be assessed, although technical briefs have been created and shared with multiple countries, and this technique could be expanded to MMN supplementation programmes if DHS questionnaires are modified to include MMN supplements.

### 3.4 Data collection systems/approaches

Monitoring and surveillance data primarily depend on routine healthcare systems (such as LMIS, HMIS, and ANC) and periodic surveys (such as DHS and MICS). The Ministry of Health management information system (MIS) collects and analyses routine health system data, such as health clinic records (e.g., patient records and programme records), growth monitoring records, and product distribution logs or supply/inventory records. The data quality from routine healthcare systems depends on the strengths and weaknesses of the MIS in a given country or context; further, the data usually represent only those who use the public health system services. There are opportunities with technology and new tools to strengthen the quality of MIS data, including indicators for MMN supplementation. For example, the District Health Information system 2 (DHIS2, http://dhis2.org/) is a flexible, Web‐based open‐source information system (software) for data collection, validation, analysis, and presentation of aggregate statistical data. DHIS2 is currently used in more than 30 countries around the world. Other sources of key indicators for MMN supplementation programmes may include periodic population‐based cross‐sectional surveys or lot quality assurance sampling monitoring systems. Depending on their design and implementation, these can provide population‐based, high‐quality, and representative data for key programme indicators. Although not representative of the population, sentinel site monitoring can potentially provide data for key indicators and track trends and changes in indicators of interest over time to help determine if MMN programmes are achieving their objectives.

### 3.5 ANC and broader health sector considerations

Access to antenatal care is the routine attention that all healthy women can expect to receive during pregnancy to improve their health and their babies' health and development. Globally, data from 2010 to 2015 shown that about 85% of pregnant women had access to ANC with a skilled health personnel at least once, but only six in 10 (58%) receive at least four ANC visits. In regions with the highest rates of maternal mortality, such as sub‐Saharan Africa and South Asia, even fewer women received at least four antenatal visits (49% and 42%; UNICEF, at https://data.unicef.org/topic/maternal-health/antenatal-care/). As such, ANC still provides an effective platform to deliver a package of maternal interventions on a large scale; therefore, indicators related to this platform are relevant to consider in the monitoring of MMN supplementation in pregnancy. The key indicator used to track improvements in maternal and reproductive health is the percentage of women 15–49 years with a live birth in a given time period who received ANC four or more times during pregnancy (ANC 4+); this percentage was one of 48 technical Millennium Development Goal indicators (United Nations, 2003). A comparable indicator has also recently been proposed under “Goal 3: Ensure healthy lives and promote well‐being for all at all ages” as part of the new United Nations Sustainable Development Goals (http://unstats.un.org/sdgs/iaeg-sdgs/metadata-compilation/)—which builds on the previous Millennium Development Goals. ANC is one of six interventions suggested for inclusion as a coverage tracer intervention to represent the broader topic of universal healthcare. The indicator is defined as number of people receiving ANC (at least four visits) divided by number of people who need ANC services. Although this indicator provides essential information on ANC access, it does not indicate the quality of care, and it may divert resources and attention from delivering and prioritizing the individual components of care. This includes commodity‐dependent interventions, such as MMN supplements, if programmes are focused primarily on achieving ANC visits, as opposed to improving quality of care. For example, a recent 41‐country analysis of DHS data found that 72% of women failed to receive 90+ IFA supplements (Hodgins & D'Agostino, 2014). To address the quality of ANC services rather than access alone, Kyei et al. proposed an indicator classifying women into a high or moderate quality‐of‐care category based on the number of services they received (Kyei, Chansa, & Gabrysch, 2012). A shortfall of this indicator is that the information needs to be obtained from a population‐based survey because HMIS systems usually do not track the number of interventions provided to each individual woman; hence, Hodgins and D'Agostino suggested an indicator based on the average coverage across services received (Hodgins & D'Agostino, 2014). When designing a composite indicator for ANC care, it is important for MMN supplementation to be considered, given the historically low success rate of supplementation programmes and the intervention's importance in improving maternal‐ and child‐health outcomes.

One major challenge to monitoring the key elements of ANC services is that systems are often established vertically and the individual components are the responsibility of different departments within the health sector (e.g., nutrition, family planning, malaria, and immunizations). This can result in fragmented programme monitoring systems that are inefficient, wasteful, and discouraging to frontline staff as they face an ever‐increasing workload (Chaulagai et al., 2005). Developing a streamlined monitoring system that makes better use of time and resources will require improved communication and coordination within the health sector. At the district level, one potential approach to promote intersectoral integration is to convene district health management meetings to coordinate ANC monitoring activities. For instance, the Indian Ministry of Health and Family Welfare recently established “integrated monitoring teams” in high‐focus districts to review action plans and discuss how to improve programme implementation and monitoring (Prasad, Chakraborty, Yadav, & Bhatia, 2013).

There are also biological reasons for establishing integrated monitoring systems in settings with widespread infections. First, impaired micronutrient absorption and metabolic alterations occur during states of acute and chronic inflammation, especially in the case of iron, where reduced iron erythropoiesis is mediated by hepcidin (Allen, 2005). Second, safety concerns have been noted around providing iron in settings with recurrent infections, particularly malaria (WHO, 2007). The current evidence indicates that as long as women receive adequate healthcare, the benefits of supplementation during pregnancy likely outweigh the risks (Sangaré, van Eijk, Ter Kuile, Walson, & Stergachis, 2014).

The WHO recommends daily IFA supplementation during pregnancy with the caveat that “in malaria‐endemic areas, provision of iron and folic acid supplements should be implemented in conjunction with measures to prevent, diagnose and treat malaria” (WHO, 2016). This recommendation may also be expected to apply to MMN supplementation; thus, integrating nutrition and malaria monitoring systems becomes advantageous. The *World Malaria Report* (2014) includes two malaria indicators that could be considered for inclusion when implementing MMN supplementation in a malaria‐endemic area: (a) percentage of pregnant women who slept under an insecticide‐treated bed net the previous night, and (b) percentage of women who received at least three or more doses of intermittent preventive treatment at ANC visits during their last pregnancy (WHO, 2014).

With the increasing recognition of the synergies between MMN supplementation and other ANC interventions, priority areas to consider for improving monitoring and surveillance include strengthening indicators for quality of services; intersectoral coordination; and, in malaria endemic areas, strengthening monitoring of malaria programmes.

## 4 DISCUSSION

In this paper, we described MMN supplementation in pregnancy indicators to be considered for programme monitoring and national and global surveillance, such as DHS and MICS. The suggested indicator titles and indicator matrix are based on the programme theory and expected intervention processes, as described in the WHO/CDC logic model (De‐Regil et al., 2014), and on practice‐based evidence from existing supplementation monitoring and implementation manuals, published literature, country reports, and the opinion of experts. When describing indicators, considerations were given for data needs at various stages of the programme lifecycle, feasibility, and cost. We considered mechanisms for collecting these data, including integration within the existing government monitoring systems, as well as other existing or potential systems. Finally, we proposed revisions to global and national surveillance DHS indicators based on these reviews.

There are many gaps in current monitoring and surveillance systems, and more than one system will often need to be used to capture the necessary programme indicators. For example, the current HMIS cannot obtain information related to intake adherence because women are not taking the actual pills at the clinic and HMIS do not currently monitor whether women reported taking pills at a later ANC visit. In DHS surveys, women are asked about the number of pills consumed during their last pregnancy, which could be up to 5 years ago, creating a major issue with recall bias. The HMIS in different countries do not necessarily follow a standard approach, and the data are asked differently across countries. A report (Dwivedi et al., 2014) from 13 sub‐Saharan Africa and south Asia countries found the majority of countries (12 of 13) did include IFA indicators in their HMIS, but much fewer include an indicator for consumption of 90 or more pills (six countries). There was also variations in whether it was captured in facility register, reported to higher levels in the facility monthly report, and/or client card. The use of HMIS and DHS data can be triangulated to get a better sense of where current programmatic bottle necks exist, but there also needs to be new and innovative approaches to collecting high‐priority indicators, such as intake adherence.

The importance of input indicators emerged as a high priority because early on the big hurdle will be building political commitment to switch from IFA to MMN supplementation. Once there is buy‐in and new guidelines and processes are in place, only small modifications will need to be made to the downstream programme indicators in countries where IFA supplementation programme previously existed. The indicators listed in this paper are useful for monitoring and surveillance, but they can also help document changes, advocate for change, propose/revise policies, and help identify where to put funding or resources.

In summary, all the indicators presented in this manuscript are useful for consideration when designing and implementing an MMN supplementation in pregnancy programme monitoring system and for surveillance in various settings. At a minimum, to ensure a successful MMN supplementation programme, most programmes should include key indicators related to the monitoring of supply, coverage, and intake adherence. The suggested monitoring indicators described in this paper are relevant for many situations, but they need to be considered with regard to local data needs and requirements. They can be adapted or other monitoring indicators considered depending on the programme context and circumstances.

## CONFLICT OF INTEREST

The authors declare that they have no conflicts of interest.

## CONTRIBUTIONS

ZM, SN, and MJ led in the concept design, data interpretation, and drafted the manuscript. PS and RFA assisted in the concept design, data interpretation, and manuscript revision. All authors read and approved the final manuscript as submitted. The findings and conclusions in this report are those of the authors and do not necessarily represent the official position of the Centers for Disease Control and Prevention and the United States Agency for International Development.

## Supporting information


**Appendix 1.** Indicator matrix by relevant indicator types and logic model categories for consideration in the monitoring and surveillance of multiple micronutrient (MMN) supplementation in pregnancy programmes.
