# Supplementary material for: Monitoring and surveillance for multiple micronutrient supplements in pregnancy
Source: Matern Child Nutr. 2017 Dec 22;14(Suppl 5):e12501. doi: 10.1111/mcn.12501 (PMC6866115; doi:10.1111/mcn.12501)
Supplement: Supplementary file 1 — Appendix 1. Indicator matrix by relevant indicator types and logic model categories for consideration in the monitoring and surveillance of multiple micronutrient (MMN) supplementation in pregnancy programmes. [file MCN-14-e12501-s001.docx]

Appendix 1. Indicator matrix by relevant indicator types and logic model categories for consideration in the monitoring and surveillance of multiple micronutrient (MMN) supplementation in pregnancy programmes

| **Corresponding WHO/CDC logic model^*^ box category** | **Indicator title** | **Calculation of indicator**  **(Operational definition)** | **Potential data collection methods / Data sources** | **Frequency of collection** | **Target** |
| --- | --- | --- | --- | --- | --- |
| **Type of indicator: Inputs** | | | | | |
| **Management, staff, national coalition, financial resources, infrastructure, other material, contributions from partners** | Key stakeholders support MMN supplements in pregnancy programme | Documented commitment of key stakeholders who support the establishment and sustainability of MMN supplementation programme in pregnancy.  Yes/No | Review documentation from key stakeholders | Annually | Yes |
|  | Work plan for forthcoming year confirms supplies, human resources, and budget:   1. MMN supplement supplies 2. Human resources 3. Budget | MMN work plan exists  Yes/No   1. work plan includes MMN supplement supply estimates   Yes/No   1. work plan includes an estimate of human resource needs   Yes/No   1. work plan includes an estimate of the required budget   Yes/No   1. work plan includes budget commitment   Yes/No | Review work plan for the next year. | Annually, or more frequently, if problems identified. | Yes |
|  | MMN supplement-related management and coordination group with defined responsibilities exists | MMN management and coordination group with defined responsibilities exists and functions according to national guidelines.  Yes/No | MMN management plan and meeting minutes for meeting schedule, roles planned, deliverables, and use of monitoring data. | Annually | Yes |
| **Type of indicator: Activities** | | | | | |
| **Policies** | National policy/guideline in place includes MMN supplements in pregnancy | Documented national policy/guideline and includes MMN supplements in pregnancy.  Yes/No | Review documentation from related government agencies | Annually until policy/guidelines in place | Yes |
| **Production and supply** | 1. Work plan exists for timely procurement of supplements 2. MMN supplement formulation procured follows national guideline | 1. Documentation of work plan exists with timeline to procure MMN supplements.   Yes/No   1. Documentation of procured MMN supplement formulation to examine whether it follows national guideline   Yes/No | Review documentation from related government agencies or related programme implementation offices | Annually | Yes |
|  | Training materials and job aids developed | Documentation of training materials and job aids exists.  Yes/No | Approved training materials and job aids | Quarterly until developed | Yes |
| **Delivery** | 1. MMN distribution system in place 2. Government agrees to include MMN in Logistic Management Information System (LMIS) and Health Management Information System (HMIS) | 1. Documentation of MMN distribution system in place   Yes/No   1. Documentation of government agreement to include MMN in Logistic Management Information System (LMIS) and Health Management Information System (HMIS)   Yes/No | Government/  facility forms include MMN in HMIS/LMIS | Quarterly until included | Yes |
|  | Supportive supervision system developed | Documentation of supportive supervision system developed  Yes/No | Review of supportive supervision work plan on file | Initially, until developed  Periodically, if warranted | Yes |
| **Quality** | 1. Quality assurance plan in place for MMN supplement programme 2. MMN monitoring plan integrated into government system to support quality control of MMN | 1. Documentation of MMN monitoring plan exists.   Yes/No   1. Documentation of MMN monitoring plan integrated into government system to support quality control of MMN   Yes/No | Quality assurance monitoring plan disseminated.  Review documentation of evidence that plan is integrated and being executed by the government. | Initially, until developed  Periodically, if warranted | Yes |
| **Behaviour change intervention (BCI)** | BCI strategy exists for MMN supplementation focused on supporting programme participants and for programme advocacy | BCI strategy document exists Yes/No | Review of finalized BCI strategy document | Initially until developed  Periodically if warranted | Yes |
| **Type of indicator: Outputs** | | | | | |
| **Availability of intervention in- country** | MMN supplements available at the national level (or central) warehouse | MMN supplements available at the national or central warehouse  Yes/No | Government delivery certificates | Annually or per procurement cycle | Yes |
| **Importation, production, and distribution of products meets quality standards and specifications** | Certificate of conformity for each shipment of imported MMN supplements | Certificate of conformity for each shipment of imported MMN supplements  Yes/No | Review of Certificates of conformity on file | Annually or per procurement cycle | Yes |
|  | Adequate storage of MMN supplements at warehouse and distribution sites | Numerator: number of monitoring reports at sites that show they are stored according to standards assessed over a given period of time (e.g., 6 months)  Denominator: number of sites assessed over same period of time | Monitoring reports/LMIS | Quarterly or every 6 months | To be defined by country |
|  | All distribution sites submit timely reports per national guidelines | Numerator: number of sites not submitting timely reports for a given period of time (e.g., last month)  Denominator: number of sites assessed for same period of time (e.g., last month) | Reports submitted/  LMIS | Monthly, quarterly | To be defined by country |
| **Providers/distributors have knowledge and motivation to adequately distribute, inform, and problem- solve with target population** | Timely training (initial and refresher) activities conducted for providers/distributors per national guidelines   1. Initial training conducted among providers/distributors per national guidelines 2. Refresher training conducted for providers/distributors per national guidelines | 1. Numerator: number of health centers where all newly hired personnel involved in distributing MMN to pregnant women have NOT received initial training per national guidelines during a given period of time (e.g., last 6 months)   Denominator: number of health centers assessed during the same period (e.g., last 6 months)   1. Numerator: number of health centers where all personnel involved in distributing MMN to pregnant women have NOT received refresher training per national guidelines during a given period of time (e.g., last 12 months)   Denominator: number of health centers assessed during the same period (e.g., last 12 months) | Health center training records/HMIS/LMIS | Per national guidelines (e.g., every 6 months for initial training and annually for refresher training) | To be defined by country |
|  | Staff who deliver MMN to pregnant women have adequate knowledge and skills to distribute and counsel | Numerator: number of staff who meet criteria for adequate knowledge and skills for a given period of time  Denominator: number of staff assessed over same period of time | Tests, training checklists, or supportive supervision checklists | During/after initial training, annually thereafter | To be defined by country |
| **Coverage of intervention** | Coverage of MMN supplements among pregnant women | Numerator: number of pregnant women who received MMN supplements during pregnancy consistent with national policy, over a given period of time;  Denominator: total number of pregnant women assessed over the same period of time. | HMIS reports  Surveys | -HMIS reporting cycle  -Periodic surveys |  |
|  | Coverage of counseling on MMN supplementation among pregnant women | Numerator: number of pregnant women who received counseling on MMN supplementation during pregnancy consistent with national policy over a given period of time;  Denominator: total number of pregnant women assessed over the same period of time. | HMIS reports  Surveys | -HMIS reporting cycle  -Periodic surveys | To be defined by country |
| **Access to or presence of intervention in communities or facilities** | 1. Sufficient MMN supply at distribution sites to cover the target population (target population denominator) 2. Distribution sites (health centers and districts level) reporting stockout in the last quarter | 1. Numerator: number of distribution sites had sufficient MMN supplements;   Denominator: total number of distribution sites.   1. Numerator: Number of health centers (or districts) reporting stock outs of MMN during the past quarter   Denominator: Total number of health centers (or districts) assessed during the last quarter | LMIS supply reports | Quarterly | To be defined by country |
| **Target population knows, demands, accepts and has ability to appropriately use the intervention** | Skills, knowledge, motivation, and attitudes about MMN supplementation among pregnant women, family decision makers, and community leaders | Numerator: number of targeted individuals who meet criteria for skills, knowledge, motivation and attitudes over a given period of time;  Denominator: total number of individuals assessed over the same period of time. | Surveys | -Periodic surveys | To be defined by country |
| **Target population uses intervention appropriately** | 1. MMN supplement intake adherence among women during pregnancy 2. Women who report consuming at least 75% of the recommended number of MMN supplements during the last pregnancy | 1. Average intake adherence   Numerator: sum of actual MMN supplements consumed as reported by pregnant women over a given period of time; Denominator: total number of MMN supplements expected to be consumed by pregnant women for the same period of time.   1. Numerator: number of women who report consuming at least 75% of the recommended number of MMN supplements during pregnancy assessed over a given period of time; Denominator: total number of women assessed for the same period of time. | Surveys | Periodic surveys | To be defined by country |
| **Type of indicator: Outcomes** | | | | | |
| **Improved nutritional status** | Iron status as measured by serum or plasma ferritin   1. Average serum or plasma ferritin concentration 2. Prevalence of low serum or plasma ferritin | 1. Numerator: sum of serum or plasma ferritin concentrations for each pregnant women assessed;   Denominator: number of pregnant women assessed.   1. Numerator: number of pregnant women below the cut-off value defined as iron deficiency. Denominator: total number of pregnant women assessed. | Surveys | Periodic surveys | To be defined by country |
|  | Folate status as measured by red blood cell folate   1. Average red blood cell folate concentration 2. Prevalence of low red blood cell folate | 1. a. Average of all pregnant women assessed 2. Numerator: sum of RBC folate concentrations for pregnant women assessed; Denominator: number of pregnant women assessed. 3. b. Numerator: number of pregnant women below the cut-off value defined as folate deficiency. 4. Denominator: total number of pregnant women assessed. | Surveys | -Periodic surveys  -HMIS and ANC reporting cycle |  |
|  | Anaemia status as measured by hemoglobin   1. Average hemoglobin 2. Prevalence of low hemoglobin | 1. Numerator: sum of hemoglobin concentrations for each pregnant women assessed; Denominator: total number of pregnant women assessed. 2. Numerator: number of pregnant women below the cut-off value defined as anaemia.   Denominator: total number of pregnant women assessed. | Surveys | Periodic surveys | To be defined by country |
|  | Urinary iodine concentration | Median urinary iodine concentration | Surveys | Periodic surveys | To be defined by country |
| **Decreased morbidity and mortality** | Low birth weight   1. Mean birth weight 2. Prevalence of low birth weight | 1. Numerator: sum of birth weight for each live birth child delivered by pregnant women; Denominator: total number of live births. 2. Numerator: number of live birth with birth weight <2500 grams. Denominator: total number of live births. | Hospital registries  HMIS and ANC reports | HMIS and ANC reporting cycle |  |
|  | Small for gestational age (SGA) | Numerator: number of live births with SGA.  Denominator: total number of live births. | Hospital registries  HMIS reports | HMIS and ANC reporting cycle | To be defined by country |
|  | Birth prevalence of neural tube defects (NTDs) | Numerator: number of births with NTDs.  Denominator: total number of births. | Hospital registries  HMIS reports | HMIS and ANC reporting cycle | To be defined by country |
|  | Pregnancy-induced hypertension (PIH) | Numerator: number of pregnant women with PIH.  Denominator: total number of pregnant women. | Hospital registries  HMIS reports | HMIS and ANC reporting cycle | To be defined by country |
|  | Maternal mortality | Numerator: number of women who died during pregnancy.  Denominator: total number of pregnant women. | Hospital registries  HMIS and ANC reports | HMIS and ANC reporting cycle | To be defined by country |

^*^ Reference (De-Regil et al. 2014)
